# Hand hygiene practice compliance among healthcare workers in a tertiary healthcare hospital in Kathmandu, Nepal

**DOI:** 10.1371/journal.pgph.0003322

**Published:** 2024-08-23

**Authors:** Sarita Duwal, Lee Budhathoki, Manisha Dhaubanjar, Durga Rijal, Puspa Acharya

**Affiliations:** 1 Department of Public Health, Nepalese Army Institute of Health Sciences, Shree Birendra Hospital, Kathmandu, Nepal; 2 Department of Community Medicine, Nepalese Army Institute of Health Sciences, Shree Birendra Hospital, Kathmandu, Nepal; 3 Iwamura College of Health Sciences, Bhaktapur, Nepal; 4 Department of Public Health, Shree Birendra Hospital, Kathmandu, Nepal; Anusandhan trust, INDIA

## Abstract

Hand hygiene is a critical practice to prevent healthcare associated infections (HCAIs). However, compliance to it among healthcare workers is very low. The study aims to assess hand hygiene practices in various situations among the healthcare workers of different departments in a tertiary care hospital in Nepal. The study is a hospital-based cross-sectional observational study among 260 healthcare workers selected using the stratified Proportionate random sampling method. Healthcare workers eligible for the study were those in ward rounds, doing procedures, and having actual contact with the patients and their surroundings. Using a WHO-developed checklist, an observation technique was used to collect the hand hygiene practice data among the healthcare workers. A total of 1068 hand hygiene opportunities were observed among 260 healthcare workers. The overall hand hygiene compliance was 30%, and the compliance was observed differently among the healthcare workers, where compliance of hand hygiene for doctors, nurses, and paramedics were 37%, 35%, and 23%, respectively. The WHO "5 moments" for hand hygiene compliance, "after body fluid exposure," was found to be higher (83%), followed by "after touching the patients’ surroundings" (79%), and only 11% compliance was found "before touching the patient." Hand hygiene procedures were missed by 36% of paramedics, 30% of doctors, and 20% of nurses. The study reflects the need to promote hand hygiene practices among healthcare workers to prevent HCAIs, as compliance with hand hygiene action was only 30%.

## Introduction

Hand hygiene (HH) is as effective as vaccines, even more than some vaccines. Despite this, hundreds of millions worldwide suffer from healthcare-associated infections (HAIs) yearly, many of which are preventable [1]. Hospital care-associated infections (HCAI) are major health safety issues worldwide and are defined as infections acquired while receiving treatment for medical or surgical conditions which were not present during the time of admission [2]. The burden of hospital-acquired infections is increasing globally and the consistent emergence of antimicrobial-resistant bacteria, particularly Gram-negative bacteria, is contributing to the challenge of HAIs including prolonged hospital stays, enduring disabilities, heightened microbial resistance to antimicrobials, substantial financial burdens on healthcare systems, increased costs for patients and their families, and elevated mortality rates [3, 4].

Extensive research findings and substantial evidence consistently demonstrate that appropriately performed hand hygiene plays a pivotal role in reducing the cross-transmission of infections within healthcare facilities (HCFs) [5, 6].

Despite the critical importance of hand hygiene in preventing healthcare-associated infections (HCAIs), healthcare workers exhibit notably low adherence to this practice [7]. A study conducted in Nepalese hospital settings revealed a relatively high level of knowledge about hand hygiene among healthcare workers; however, this knowledge did not consistently translate into actual practices [8]. Monitoring hand hygiene practices in healthcare facilities is imperative. The direct observation method, often regarded as the "Gold Standard," is considered one of the most effective ways to assess compliance. This method, simple yet cost-effective, serves as a reliable measure of compliance [9]. The study’s objective is to evaluate hand hygiene practices in diverse situations among healthcare workers from different departments.

## Methods

The study was a cross-sectional study conducted within the institutional setting of Shree Birendra Hospital, Kathmandu, involving 260 healthcare workers. The study was conducted from November 15 to December 30, 2021 including different departments.

### Ethics statement

The study was conducted from November 15 to December 30, 2021 including different departments. Ethical approval was obtained from the Institutional Review Committee (IRC) of Nepalese Army Institute of Health Sciences (NAIHS) dated on October, 2021 with IRC No. 474. Original copy of the ethical approval is submitted as the S1 Data. Since, the study was observational study; informed consent was not obtained from the participants. However, the study maintained the anonymity of the participants.

### Measures

A single observer was trained on the correct method of monitoring hand hygiene (HH) by a nurse specialized in Infection Prevention and Control (IPC). The observer practiced the technique alongside the IPC nurse before officially collecting data to ensure consistency in data collection. The study focused on healthcare workers engaged in rounds, procedures, and direct patient contact, observing each participant for a minimum of 20 minutes during each session. Observations were conducted during the morning shift, specifically from 8 am to 4 pm.

The hand hygiene compliance checklist developed by the World Health Organization (WHO) was employed for data collection [10]. Subsequently, data entry was performed using EPI data version 3.1, and the data were exported to SPSS version 16 for analysis. The compliance rate was calculated by using the formula:

Compliance (%) = Actions/Opportunities x 100

### Sample size and sampling techniques

The Stratified Proportionate Sampling Technique was applied to select a representative sample of 260 individuals from a total of 520 hospital staff present during the morning shift. Healthcare workers eligible for the study were those in ward rounds, doing procedures, and having actual contact with the patients and their surroundings. The staff was categorized into three strata: doctors (140), nurses (150), and paramedics (Medical Assistant) (230). Proportions of each stratum were calculated, and sample sizes were determined by multiplying these proportions by the desired total sample size. The resulting sample sizes were approximately 70 for doctors, 75 for nurses, and 115 for paramedics; that is, 260 samples. The final step involved randomly selecting individuals from each stratum by using a lottery method to represent the diverse roles within the hospital staff accurately. After completing the data collection, data was checked for errors in Microsoft Excel and calculation was done.

Calculation:

Proportion of doctors: 140/520 = 0.2692

The desired sample size for doctors: 0.2692×260 = 70.23 ≈ 70

Proportion of nurses: 150/520 = 0.2885

The desired sample size for nurses: 0.2885×260 = 75.01 ≈ 75

Proportion of paramedics: 230/520 = 0.4423

The desired sample size for paramedics: 0.4423×260 = 114.96≈ 115

## Results

During the study, 1068 HH opportunities (for five moments of hand hygiene) were observed. Among 260 HCWs, the majority of them were paramedics 115 (44%), followed by nurses 75 (28.8%), and doctors 70 (26.9%).

The overall compliance was 30%, out of which doctors’ compliance was 37%, followed by nurses at 35% and only 23% for paramedics. Among various HCWs, 36% of paramedics, 30% of doctors, and 20% of nurses missed the HH practices. The practice of alcohol-based hand rubs 163 and hand washing 159 were nearly equal (Table 1).

**Table 1:** Hand hygiene compliance among different professions (Total observation, N = 1068).

Professions	Hand Hygiene (HH) Action				Total	
	Hand Rub (HR)	Hand Wash (HW)	Missed	Gloves	Opportunities	Compliance (%)
Doctor	52	24	62	68	206	37
Nurse	68	72	79	181	400	35
Paramedics	43	63	168	188	462	23
Total	163	159	309	437	1068	30

The most common indication for HH action was "before touching the patient" (43%), and the action performed was using gloves. The highest number of missed HH actions occurred during moment 1 (before touching the patient), with 156 (34%) instances missed out of 457 occasions. During moment 2 (before the clean/aseptic procedure), every HCW didn’t miss a single HH action. Compliance with the 5 moments of HH varied according to the indications for HH, from a highest of 83% after body fluid exposure, followed by 79% after touching the patient, to the lowest of 11% before touching the patient (Table 2).

**Table 2:** Summary of common indications, hand hygiene actions, and their compliance (Total observation, N = 1068).

Indication	Hand Hygiene (HH) Action				Total	
	Hand Rub (HR)	Hand Wash (HW)	Missed	Gloves	Opportunities	Compliance (%)
Before touching a patient	31	18	156	252	457	11
Before the clean/aseptic procedure	4	51	0	22	77	71
After body fluid exposure	13	46	3	9	71	83
After touching patient	54	35	7	16	112	79
After touching patient’s surrounding	61	9	143	138	351	20
Total	163	159	309	437	1068	30

In this study, doctors showed 100% compliance during moment 2 (before clean/aseptic procedure) and moment 3 (after body fluid exposure). For nurses, the compliance rate was higher (86%) during moment 3 (after body fluid exposure) followed by moment 4 (after touching the patient), while paramedics showed higher (85%) compliance during moment 4 (after touching the patient).

## Discussions

In the healthcare setting, Pittet (2000) established that maintaining hand hygiene is the foremost measure to prevent infection transmission [11]. Healthcare providers must consistently follow hand hygiene protocols before and after patient contact, preceding and following procedures, and while handling patients’ surroundings. Adhering to these critical points is essential for disrupting the transmission of microorganisms and plays a vital role in infection control.

This study found that hand hygiene compliance was 30%, that is much lower than similar studies conducted in Nepal (52%) and India (46.1%) [8, 12]. About one-third of participants followed hand hygiene, which is slightly better than another study in Kathmandu (24.25%) [8]. In Nigeria, compliance was 48.2% [13]. China and Kuwait reported similar rates of compliance 17.44% and 25%, respectively [14, 15]. Possible reasons for these differences might include varying health care practices, policies, and resources in different regions. Cultural practices and community awareness could also play a role for the differences in compliance. Despite these variations, challenges like high workload, time constraints, and limited awareness may contribute to lower the global hand hygiene compliance adherence. Modified interventions that take into account the specific challenges of each region are essential for improving practices.

Among healthcare professionals, the study found that doctors exhibited a hand hygiene compliance rate of 37%, nurses had a rate of 35%, and paramedics demonstrated a lower compliance rate of 23%. This contrasts with the findings of Onyedibe, et al. [13], who reported higher compliance among nurses (36%) than doctors (31%). However, Chavali et al. [16] reported contrasting result that allied staff showed the highest compliance (86.9%) than nurses (69%). Group specific training programs, institutional policies, and job duties may contribute to the observed differences.

Within the healthcare worker categories, 36% of paramedics, 30% of doctors, and 20% of nurses were noted to have missed practicing hand hygiene (Table 1). These figures were significantly lower than the results of Semwal et al., [17], which found that 63% of doctors and 62% of nurses missed hand hygiene practices. Regarding hand hygiene methods, alcohol-based hand rub was observed in 15% of cases, whereas hand washing was observed in 14.9% of cases. This distribution is similar to the previous study [17], where there was just a 1% difference in the practice of hand washing (20%) and hand rubbing (21%). However, a study in another tertiary care hospital in Nepal found a difference between the use of alcohol-based hand rub (36.5%) and hand washing (15.4%) [5].

Our study found that the most common reason for healthcare workers to practice hand hygiene (HH) was "moment 3" (After body fluid exposure) which accounted for 83% of observed instances (Table 2). This practice was followed by "moment 4" (after touching patients) that differs from previous study [17]. This finding aligns with a study by Labi et al., [18], who reported the highest compliance during moments 3 and 4. The study highlights that healthcare staff were particularly careful about hand hygiene after dealing with body fluids; this could be due to the realization of importance to protect themselves. On the other hand, compliance was notably low (20%) for "moment 5" (after touching patient surroundings) (Table 2). This is consistent with findings from Onyedibe, et al., [13]; however, the results differ from another study [19] regarding poor compliance for "moment 5" across all staff.

Doctors exhibited full compliance, up to 100%, during "moment 2" and "moment 3" (Table 3). In contrast, nurses and paramedics demonstrated a higher compliance rate (86%) during "moment 3" and "moment 4". This inclination might be influenced by the prevailing belief that healthcare workers are primarily motivated by self-protection [20].

**Table 3:** Summary of hand hygiene compliance based on indication and profession (Total observation, N = 1068).

Indication	Compliance (%)		
	Doctor	Nurse	Paramedics
Before touching a patient	17	13.4	5
Before the clean/aseptic procedure	100	78	58
After body fluid exposure	100	86	77
After touching patient	68	81	85
After touching patient’s surrounding	41	20	11

The most significant lapse in hand hygiene actions was observed during "moment 1", where 156 missed opportunities were noted out of 457 observations (34%) (Table 3). This pattern coincides with the findings of Semwal et al., [17] suggesting a consistent trend across healthcare settings. Possible reasons for this observed lapse could include time constraints, inadequate awareness, and / or the perceived urgency of the situation. Addressing these factors may be essential in developing effective strategies to improve hand hygiene practices, particularly before direct patient contact.

### Strengths and limitation

The study was conducted in a single hospital setting; thus, it is important to confirm our findings with the further studies to generalize across the health care settings. Moreover, the availability of hand washing facilities in the hospital could impact the observed hand hygiene practices. Using the observation technique may also introduce a Hawthorne effect. To reduce the effect we assigned a single observer during data collection. Despite the limitations of this study, our findings give a glance regarding the hand hygiene practices among healthcare workers in Nepal health care settings. We believe this study will be an important baseline evidence for strengthening IPC initiatives and ensuring patient safety.

## Conclusions

In summary, this study highlights only 30% of compliance rate of hand hygiene practices existed among health care workers. This poses a serious threat to Infection Prevention and Control (IPC) programs. To address this challenge, continuous training, performance feedback, verbal reminders, and the provision of adequate hand hygiene facilities are essential for enhancing compliance to hand hygiene practices.

Furthermore, future research endeavors should prioritize to explore the barriers and facilitators influencing hand hygiene practices. This in-depth analyses can inform targeted intervention programs, effectively address specific challenges and foster a culture of consistent hand hygiene compliance among healthcare workers. Our findings provide an overview of hand hygiene practices among healthcare workers in Nepalese healthcare settings. We believe this study will serve as essential baseline evidence for enhancing IPC initiatives and ensuring patient safety.

## Supporting information

S1 Data (XLS)
